# Prognostic discrimination of subgrouping node-positive endometrioid uterine cancer: location *vs* nodal extent

**DOI:** 10.1038/bjc.2011.336

**Published:** 2011-09-13

**Authors:** D S Kapp, T K Kiet, J K Chan

**Affiliations:** 1 Department of Radiation Oncology, Stanford University School of Medicine, Cancer Center, 875 Blake Wilbur Drive, Stanford, CA 94305, USA; 2 Division of Gynecologic Oncology, Department of Obstetrics, Gynecology and Reproductive Sciences, University of California, San Francisco School of Medicine, Helen Diller Family Comprehensive Cancer Center, 1600 Divisadero Street, San Francisco, CA 94143, USA

**Keywords:** uterine cancer, positive lymph nodes, substaging, node location, node ratio

## Abstract

**Background:**

The 2009 International Federation of Gynecologists and Obstetricians elected to substage patients with positive retroperitoneal lymph nodes as IIIC 1 (pelvic lymph node metastasis only) and IIIC 2 (paraaortic node metastasis with or with positive pelvic lymph nodes). We have investigated the discriminatory ability of subgrouping patients with retroperitoneal nodal involvement based on location, number, and ratio of positive nodes.

**Methods:**

For 1075 patients with stage IIIC endometrioid corpus cancer abstracted from the Surveillance, Epidemiology, and End Results databases for 2003–2007, Kaplan–Meier analyses, Cox proportional hazard models, and other quantitative measures were used to compare the prognostic discrimination for disease-specific survival (DSS) of nodal subgroupings.

**Results:**

In univariate analysis, the 3-year DSS were significantly different for subgroupings by location (IIIC 1 *vs* IIIC 2; 80.5% *vs* 67.0%, respectively, *P*=0.001), lymph node ratio (⩽23.2% *vs* >23.2% 80.8% *vs* 67.6% *P*<0.001), and number of positive lymph nodes (1, 2–5, >5; 79.5, 75.4, 62.9%, *P*=0.016). The ratio of positive nodes showed superior discriminatory substaging in Cox models.

**Conclusion:**

Subgrouping of stage IIIC patients by the ratio of positive nodes, either as a dichotomized or continuous parameter, shows the strongest ability to discriminate the survival, controlling for other confounding factors.

Uterine cancer is the most common pelvic gynaecologic malignancy in the United States. Based on a Gynaecologic Oncology Group study of surgical staging of clinical stage I endometrial cancer, 9.3% of patients had positive pelvic lymph node involvement whereas 5.5% had positive paraaortic lymph nodes, with a total of 11.3% having either pelvic and/or paraaortic retroperitoneal metastasis (Creasman *et al*, 1987). The most recent modification of the International Federation of Gynecologists and Obstetricians (FIGO) staging system for endometrial cancer has elected to subclassify patients with retroperitoneal lymph node involvement (without other sites of distant metastasis) into two subgroups based on the location of the metastatic lymph nodes. Patients with only pelvic lymph node involvement are staged as IIIC 1 whereas those with positive paraaortic lymph node (with or without positive pelvic lymph nodes) are stage IIIC 2 (Pecorelli, 2009). Two recent Surveillance, Epidemiology, and End Results (SEER)-based analyses have demonstrated worse outcome for patients with stage IIIC 2 *vs* IIIC 1 disease (Lewin *et al*, 2010; Cooke *et al*, 2011). However these studies were limited because there was no accounting for confounders such as number of positive nodes (Touboul *et al*, 2001; Takeshima *et al*, 2006; Fujimoto *et al*, 2009) or lymph node ratio, which have also been shown to be prognostically important (Tang *et al*, 1998; Mariani *et al*, 2001a; Yasunaga *et al*, 2003; Chan and Kapp, 2007; Chan *et al*, 2007).

In this current study, we investigated the prognostic significance of the new subdivision of stage IIIC disease and compared the discriminatory ability of location, number, and ratio of positive lymph nodes controlling for other confounding factors. The identification of other subgrouping based on characteristics of lymph node involvement may have therapeutic implications.

## Materials and methods

The SEER Program database of the United States National Cancer Institute for endometrioid uterine cancer patients during the period from 1 January 2004 to 31 December 2007 was utilised (SEER, 2010, http://www.seer.cancer.gov). Patients with non-endometriod histologies were excluded. This time period was selected because in earlier periods patients with involved paraaortic lymph nodes were included with patients with stage IV disease. Of the 22 907 patients, 1235 (5.4%) had IIIC disease. A total of 160 patients who lacked information on lymph node dissection and/or lymph node distribution were excluded, leaving 1075 patients as the study cohort. All but four patients underwent some type of hysterectomy (three had no hysterectomy and for one the type of uterine surgery was not specified).

Data on demographic, clinical-pathological, and treatment parameters were abstracted. Patients were divided into nodal subgroups based on the number of positive nodes (1, 2–5, and >5 nodes), total number of nodes examined (⩽10, 11–20, >20), and ratio of positive nodes, expressed as the percentage of positive lymph nodes to the total number of nodes examined (⩽10%, 10–50%, >50%), as in our previous study (Chan *et al*, 2007). In addition, the nodal parameters were dichotomized by the median number of positive nodes (1, >1) and ratio of positive nodes (⩽ or >average (23.2%)) to permit comparison with the new FIGO dichotomized stage grouping.

The primary endpoint of the study was the endometrial cancer disease-specific survival (DSS). Time to death was censored in patients who died from causes other than uterine cancer. Survival analyses were performed using the Kaplan–Meier method. Pearson's *χ*^2^- and Student's *t*-test were employed to compare distributions of parameters between subgroups. Two-sided *P*-values of <0.05 were considered statistically significant. Pearson correlations were used to investigate for multiple colinearities between the subgrouping of lymph nodal involvement based on location, number of positive nodes, and ratio of positive nodes. Because of the potential correlation between the various subgroupings of lymph nodes, separate stepwise Cox regression models were employed entering only one of the three subgroupings of the lymph nodes in each model. Because preliminary analysis demonstrated a significantly higher number of positive lymph nodes for patients with IIIC 2 *vs* IIIC 1 disease, comparisons were also made between the three nodal subgroupings for a subset of 487 patients with only one positive lymph node. Three additional quantitative measures were used to compare the prognostic discrimination for DSS for nodal subgrouping (Gimotty *et al*, 2005). All statistical analyses were performed using the SPSS Statistics GradPack 17.0, Release 17.0.0 (3 August 2008, IBM, Armonk, NY, USA).

## Results

The demographic and clinical characteristics of the 1075 patients with stage IIIC endometrioid corpus cancers are delineated in Table 1. A total of 725 patients (67.4%) had positive pelvic nodes only (stage IIIC 1) whereas 350 (32.6%) had paraaortic node involvement with or without positive pelvic nodes (stage IIIC 2). The average number of lymph nodes examined was 17.3 (range: 1–90). The average number of positive nodes was three (range: 1–82) and the average lymph node ratio was 23.2% (range 0.01–100%). Adjuvant radiation therapy was given to 638 (59.3%) of the patients. The median follow-up was 18 months (mean 19.4, range 0–47).

**Figure 1:**
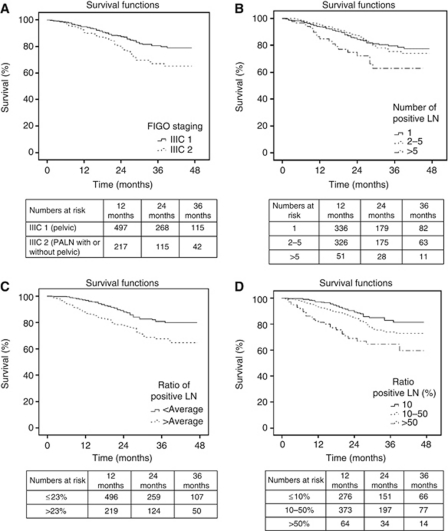
Kaplan–Meier disease-specific survival of stage IIIC endometrioid uterine cancer based on the following: (**A**) FIGO stage IIIC 1 *vs* IIIIC 2; *P*=0.001. (**B**) Number (1 *vs* 2–5 *vs* >5) of positive lymph nodes; *P*=0.016. (**C**) Ratio of positive nodes (⩽23.2% *vs* >23.2%), *P*<0.001. (**D**) Ratio of positive nodes (⩽10% *vs* 10–50% *vs* >50%); *P*<0.001.

In univariate analysis, race (Black and unknown or others, *P*=0.042), higher grade (*P*<0.001), lack of adjuvant radiation therapy (*P*<0.001), not married patients (*P*<0.005), and nodal parameters including lower number of lymph nodes examined (*P*=0.001), higher number of positive lymph nodes (*P*=0.016), higher ratio of positive lymph nodes (*P*<0.001) and FIGO substage (stage IIIC 2 *vs* IIIC 1, *P*=0.001) were significantly associated with poorer DSS (Table 2). The 3-year DSS for stage IIIC 1 was 80.5% *vs* 67.0% for IIIC 2 (*P*=0.001; Figure 1A). The 3-year DSS for 1, 2–5 or >5 positive nodes were 79.5%, 75.4%, and 62.9%, respectively (*P*=0.016, Figure 1B). Based on ratio of positive lymph nodes to total lymph nodes examined (average=23.2%), the 3-year DSS was 80.8% for ⩽average ratio *vs* 67.6% >average ratio (*P*<0.001; Figure 1C). For patients divided into three groups based on lymph node ratio (10%, 10–50%, and >50%), the 3-year DSS decreased with increasing lymph node ratio (82.9%, 73.9%, and 64.5% respectively, *P*<0.001, Figure 1D).

Significantly poorer DSS was seen in higher grade tumours (*P*<0.001) and with the lack of adjuvant radiation (*P*<0.001). In a separate analysis (data not shown) the patients were grouped by number of lymph nodes examined (<10, 10–20, ⩾20). Even for the subgroups with ⩾20 nodes examined, significantly lower DSS was seen with increasing number of positive nodes, stage III C2 *vs* stage III C1, and higher ratio of positive nodes (*P*<0.001).

Stage IIIC 2 patients had higher number of positive nodes (*P*<0.001), higher ratio of positive nodes (*P*<0.001), and a higher number of reported lymph nodes (*P*<0.0001) than for stage IIIC 1 patients (Table 3).

On multivariate analysis, grade of tumour (*P*<0.001), ratio of positive nodes (*P*=0.005), adjuvant radiation (*P*<0.001), and marital status (*P*<0.01) were independent factors associated with DSS whereas location of the positive nodes, number of positive nodes, and number of nodes examined were not significant (Table 4). The hazard ratio for the ratio of positive nodes as a continuous variable was 3.10.

Pearson correlation coefficients were employed to look for colinearity between the subgroupings of positive lymph nodes (location, number of positive nodes, and node ratios) and showed small correlations between these variables (0.106, 0.263, and 0.321, respectively). Repeat multivariate analyses were performed by entering only one of the three lymph node parameters with age, marital status, grade, and adjuvant radiation therapy and showed all three nodal parameters were statistically significant. The ratio of positive lymph nodes as a continuous variable had the highest hazard ratio of 4.22, *P*<0.001, compared with the hazard ratio for the location of positive nodes (1.49) and number of positive nodes (1.05).

**Figure 2:**
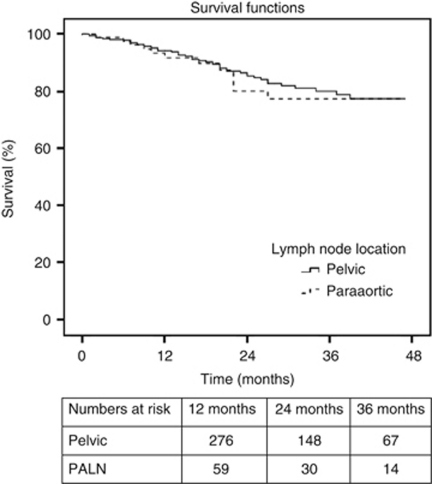
Kaplan–Meier disease-specific survival for stage IIIC endometrioid cancer patients (*n*=487) with only one positive node based on lymph node location (pelvic *vs* paraaortic); *P*=0.675.

A subset analysis was performed on those patients with one positive node (*n*=487); of which 394 (81.7%) had one positive pelvic node whereas 89 (18.3%) had one positive paraaortic node (Supplementary Table S1). There was no significant difference in 3-year DSS for patients with one positive pelvic node compared with those with one positive paraaortic node (80% *vs* 77.3%, respectively, *P*=0.675, Figure 2). These findings were confirmed on multivariate analysis (Supplementary Table S2).

The relative discriminatory ability of subgrouping patients with IIIC disease by location (IIIC 1 *vs* IIIC 2, number of positive nodes (1 *vs* >1), and ratio of positive nodes (⩽23.2% *vs* >23.2%) is shown in Supplementary Table S3. The hazard ratios for the ratio of positive nodes was 2.20, location (IIIC 1 *vs* IIIC 2) was 1.72 and 1 *vs* >1 positive nodes 1.15 (Table 5).

## Discussion

Uterine cancer is the most common gynaecologic malignancy in the United States with 43 470 new cases and 7950 deaths expected for 2010 (Jemal *et al*, 2010). There has been an increase in the number of deaths, particularly in those with advanced stage (III/IV) disease (Ueda *et al*, 2008). Although the majority have excellent prognosis with 5-year survival rates between 80–91%, the ∼5–10% of patients presenting with retroperitoneal lymph node involvement have inferior survival (Creasman *et al*, 2006; Lewin *et al*, 2010). Those with stage IIIC disease have survivals ranging from 10% to 75% (Chan *et al*, 2007). This, in part, reflects the heterogeneity in nodal and other prognostic parameters in stage IIIC cancers. The recent revision of FIGO staging of endometrial cancer has subdivided retroperitoneal node-positive patients into two subgroups based solely on the location of the positive nodes (Pecorelli, 2009).

Prior studies have shown a wide variation in survival based on lymph node location. In several series patients with involvement of pelvic lymph nodes only had nonsignificant differences in survival compared with those with positive paraaortic lymph nodes (McMeekin *et al*, 2001; Mariani *et al*, 2002; Otsuka *et al*, 2002; Havrilesky *et al*, 2005; Hoekstra *et al*, 2009; Lewin *et al*, 2010; Todo *et al*, 2011). In other series better survival was noted for patients with pelvic nodes (Morrow *et al*, 1991; Hirahatake *et al*, 1997; Yokoyama *et al*, 1997; Watari *et al*, 2005; Fujimoto *et al*, 2007; Karube *et al*, 2010; Lewin *et al*, 2010). Furthermore one recent series has shown a better survival for patients with positive paraaortic lymph nodes compared with those with positive pelvic lymph nodes (80% *vs* 55% at 5 years), although the difference was not statistically significant (Klopp *et al*, 2009). However, many of these studies did not account for the number of positive and ratio of positive nodes in addition to the location of positive nodes. Our current study analysed 1075 patients with endometrioid uterine cancer to confirm the independent prognostic significance of this new subgrouping based on node location. In addition, we compared the prognostic discrimination of nodal location with the number of positive and ratio of positive nodes.

Nodal location was found to be a significant prognostic factor both in univariate (Table 2, Figure 1A) and multivariate analysis when it was entered as the only term relating to the lymph nodes. However, the new subgrouping by nodal location was not shown to be statistically significant in multivariate analysis when the number of positive nodes and ratio of positive nodes were included (Table 4). As patients in our study with stage IIIC 2 disease had a higher number of positive lymph nodes than those with stage IIIC 1 disease (4.2 *vs* 2.0, respectively, *P*<0.001) we performed a separate analysis for those with only one positive retroperitoneal lymph node. This subgroup analysis showed no significant difference in DSS based on nodal location (Figure 2), while the ratio of positive nodes remained significant (Supplementary Table S2).

Our findings on the prognostic significance of the number of positive lymph nodes in univariate analysis (Figure 1B) confirm previous reports (Morrow *et al*, 1991; Takeshima *et al*, 1994; Touboul *et al*, 2001; Chan *et al*, 2007). Watari *et al* (2005) demonstrated a better 5-year survival rate for patients with one positive paraaortic lymph node group compared with those with ⩾2 positive paraaortic lymph node groups (60.4% *vs* 20.0%, respectively, *P*=0.0319) whereas Fujimoto *et al* (2009) reported better 5-year relapse-free survival for patients with one positive pelvic lymph node site compared with those with ⩾2 positive sites (81.3% *vs* 41.2%, respectively, *P*=0.04). We also showed the prognostic significance of the ratio of positive nodes to the total number of lymph nodes examined, which confirms our prior report (Chan *et al*, 2007). In the current study, the ratio of positive nodes was significant whether it was entered as a continuous variable in multivariate analysis, dichotomized at the mean of 23.2%, or subgrouped based on ⩽10%, 10–50%, or >50% involvement. The results from single institutional respective studies have also demonstrated the prognostic significance of ratio of positive lymph nodes (Tang *et al*, 1998; Mariani *et al*, 2001b).

Studies in other malignancies have also attempted to define the most prognostically significant subgroupings for lymph node positive patients. Various classification schemes for lymph nodes in gastric cancer have been based on the distance, number, and anatomical location of metastatic nodes as well as the site of the primary tumour (Kajitani, 1981; Hermanek and Sobin, 1992; Adachi *et al*, 1995; Sobin and Wittekind, 1997; de Manzoni *et al*, 1999). Classification of involved regional lymph nodes in gastric cancer by the ratio of positive nodes was found to represent a simple, reliable, and reproducible staging system (Yu *et al*, 1997; Liu *et al*, 2007; Marchet *et al*, 2007; Persiani *et al*, 2008; Zhang *et al*, 2009; Maduekwe *et al*, 2010; Sianesi *et al*, 2010).

The major shortcoming of any substaging of endometrial cancer based on measurements of nodal involvement is the lack of standardisation of the lymphadenectomy. There is wide variation in the extent of nodal dissection reflecting both surgeon's bias and patient selection. For example, this could include performing a more limited lymph node dissection following a resection of an involved bulky node, or performing a more extensive nodal dissection in patients without bulky nodes or co-morbidities (Smith *et al*, 2008). The issue of standardisation of lymph node dissection has been thoroughly reviewed previously (Boronow, 1980; Kilgore *et al*, 1995; Chan and Kapp, 2007; Chang *et al*, 2008; Mariani *et al*, 2009). As discussed by Mariani *et al* (2009) in their commentary on the surgical staging of endometrial cancer, a standardisation of lymphadenectomy including the anatomical extent of the paraaortic lymph node dissection is lacking. The minimum requirement of lymphadenectomy, either in terms of nodal stations resected or total number of lymph nodes examined, has not been unambiguously defined in the FIGO staging system. Recommendations as to the minimum number of lymph nodes examined for adequate nodal staging have been in effect for colon cancer (12 nodes) (Nelson *et al*, 2001) and gastric cancer (15 nodes) (Green *et al*, 2010). We are in agreement with the NCCN guidelines for the treatment of uterine cancer recommending a complete pelvic and paraaortic lymphadenectomy (unless technically unfeasible or medically contraindicated), adhering to the ACOG surgical policy (ACOG, 2005).

Two prospective randomized trials have failed to demonstrate a survival advantage from pelvic lymphadenectomy in endometrial cancer (Benedetti Panici *et al*, 2008; Kitchener *et al*, 2009). However the inclusion of low-risk patients, lack of standardisation of systemic postoperative treatments, and minimal or lack of paraaortic lymphadenectomy are limitations of these studies (Amant *et al*, 2009; Uccella *et al*, 2009; Seamon *et al*, 2010). A recent retrospective study in patients with stage III C endometrial cancer demonstrated the therapeutic significance of systematic lymphadenectomy including both pelvic and paraaortic node dissection (Todo *et al*, 2011).

Additional limitations of our study include the lack of information on other patient and treatment factors that may be of prognostic significance in patients with retroperitoneal node involvement. In particular, there is a lack of information on the extent of the pelvic and/or paraaortic lymphadenopathy, the extent of surgical staging, the surgeon's subspecialty, the extent of lymph node debulking, involvement of other pelvic extrauterine sites including the adnexa and peritoneal cytology, involvement of the uterine cervix, depth of myometrial invasion, lymph vascular space invasion, and size of the lymph nodes. Our study was limited to patients with endometrioid histology, relatively short follow-up, and there was no central pathology review. There is also a lack of information on sites of recurrence and the use of adjuvant systemic chemotherapy and hormonal therapy. However, the recent years of diagnosis of the patients included in this study should make them more likely to have received adjuvant treatment with chemotherapy or volume-directed radiation therapy and chemotherapy than studies including earlier cohorts of SEER patients. Other general limitations of SEER-based research including variation in data registry, underreporting of radiation therapy, lack of details on adjuvant radiation therapy (fields treated and doses), and selection bias have recently been reviewed by Yu *et al* (2009).

The strengths of our analysis include the large number of recently diagnosed patients with node-positive endometrioid uterine cancers studied within a wide geographic distribution in the United States. In addition, our univariate and multivariate analysis of the three major subgroupings of stage IIIC patients (based on the new FIGO substaging, number of positive lymph nodes reported, and lymph node ratio) has permitted identification of the subgroupings with better abilities to discriminate DSS in this heterogeneous group of stage IIIC patients.

In summary, better classification of retroperitoneal lymph node-positive endometrioid uterine cancer patients may permit the identification of more homogenous subgroupings for prognostic purposes, stratification in clinic trials, and possible better selection for individualised adjuvant-combined modality treatments (Mariani *et al*, 2004). Higher risk subgroups, for example those with multiple pelvic and paraaortic nodal involvement, may require more intense chemotherapy regimens, whereas those with limited nodal disease may best be managed with volume-directed radiation therapy and less toxic systemic treatment protocols. Our study has confirmed the value of subgrouping stage IIIC patients based on nodal location, number of positive lymph nodes and ratio of positive nodes. However based on multivariate and discrimination analyses, nodal ratio was a stronger discriminator for DSS than nodal location, controlling for other confounding factors including tumor grade and the use of adjuvant radiation therapy. If our results are validated in other patient databases, these findings may permit better modifications of the substaging of retroperitoneal lymph node positive patients. However, it is stressed that standardisation of lymphadenectomy including the boundaries of resection, uniform processing of the nodal specimens, and the criteria for adequacy of lymph node resection are needed.

## Figures and Tables

**Table 1:** Patient demographic and clinical characteristics

**Parameters**	***N* (%)**
*Age at diagnosis (average: 62, range: 28–95) (years)*	
<62	515 (47.9)
⩾62	560 (52.1)
	
*Marital status* a	
Married	538 (51.0)
Not married	516 (49.0)
	
*Race*	
White	917 (85.3)
Black	68 (6.3)
Asian	79 (7.3)
Unknown or other	11 (1.0)
	
*Grade*	
1	169 (15.7)
2	429 (39.9)
3	397 (36.9)
Unknown	80 (7.4)
	
*Location of positive regional nodes*	
Pelvic (stage IIIC 1)	725 (67.4)
Paraaortic with or without pelvic (stage IIIC 2)	350 (32.6)
	
*Number of positive nodes (average: 3, range: 1–82)*	
1	487 (45.3)
2–5	491 (45.7)
>5	97 (9.0)
	
*Total number of nodes examined (average: 17.3, range: 1–90)*	
⩽10	376 (35.0)
11–20	346 (32.2)
>20	353 (32.8)
	
*Ratio of positive nodes (average: 23.2%, range: 0.01–100%)*	
⩽Average	729 (67.8)
>Average	346 (32.2)
	
*Ratio of positive nodes (average: 23.2%, range: 0.01–100%)*	
⩽10%	402 (37.4)
10–50%	567 (52.7)
>50%	106 (9.9)
	
Adjuvant radiation	
No	437 (40.7)
Yes	638 (59.3)

a Marital status: total *N*=1054 due to unknowns; not married includes single, divorced, separated, and widowed.

**Table 2:** Patient parameters associated with disease-specific survival

**Parameters**	**3-year DSS (%)**	***P*-value**
*Age (average: 62, range: 28–95) (years)*		0.078
<62	79.7±2.7	
⩾62	73.2±2.9	
*Marital status* a		0.005
Married	80.4±2.7	
Not married	72.0±3.0	
*Race*		0.042
White	77.6±2.1	
Black	57.0±9.0	
Asian	79.4±7.5	
Unknown or other	66.7±27.2	
*Grade*		<0.001
1	89.8±3.5	
2	81.9±3.0	
3	63.1±3.6	
Unknown	84.7±5.2	
*FIGO stage*		0.001
IIIC 1 (pelvic)	80.5±2.2	
IIIC 2 (PALN with or without pelvic)	67.0±4.0	
*Number of positive nodes (average: 3, range: 1–82)*		0.416
1	79.5±2.6	
>1	73.4±2.9	
*Number of positive nodes (range: 3, range: 1–82)*		0.016
1	79.5±2.6	
2–5	75.4±3.2	
>5	62.9±7.3	
	*Total number of nodes examined (average: 17.3, range: 1–90)*	0.001
⩽10	70.1±3.3	
11–20	81.7±3.3	
>20	78.2±3.6	
	*Ratio of positive nodes (average: 23.2%, range: 0.01–100%)*	<0.001
⩽Average	80.8±2.3	
>Average	67.6±3.6	
	*Ratio of positive nodes (average: 23.2%, range: 0.01–100%)*	<0.001
⩽10	82.9±2.9	
10–50	73.9±2.9	
>50	64.5±5.9	
*Adjuvant radiation*		<0.001
No	67.0±3.7	
Yes	81.5±2.3	

Abbreviations: DSS=disease-specific survival; PALN=paraaortic lymph nodes.

a Marital status: total *N*=1054 due to unknowns; not married includes single, divorced, separated, and widowed.

**Table 3:** Comparison of characteristics between stage IIIC 1 and IIIC 2 patients

**Parameters**	**Stage IIIC 1**	**Stage IIIC 2**	***P*-value**
	*Age (average: 62, range: 28–95) (years)*		0.410
<62	341 (47.0%)	174 (49.7%)	
⩾62	384 (53.0%)	176 (50.3%)	
	*Marital status* a		0.957
Married	362 (51.0%)	176 (51.2%)	
Not married	348 (49.0%)	168 (48.8%)	
	*Race*		0.517
White	619 (85.4%)	298 (85.1%)	
Black	50 (6.9%)	18 (5.1%)	
Asian	49 (6.8%)	30 (8.6%)	
Unknown or other	7 (1.0%)	4 (1.1%)	
	*Grade*		0.008
1	118 (16.3%)	51 (14.6%)	
2	304 (41.9%)	125 (35.7%)	
3	243 (33.5%)	154 (44.0%)	
Unknown	60 (8.3%)	20 (5.7%)	
	*Number of positive nodes (range: 3, range: 1–82)*		<0.001
1	398 (54.9%)	89 (25.4%)	
2–5	303 (41.8%)	188 (53.7%)	
>5	24 (3.3%)	73 (20.9%)	
*Total number of nodes examined (average: 17.3, range: 1–90)*	<0.001		
⩽10	272 (37.5%)	104 (29.7%)	
11–20	246 (33.9%)	100 (28.6%)	
>20	207 (28.6%)	146 (41.7%)	
*Ratio of positive nodes (average: 23.2%, range: 0.01–100%)*	0.001		
⩽Average	515 (71.0%)	214 (61.1%)	
>Average	210 (29.0%)	136 (38.9%)	
*Ratio of positive nodes (average: 23.2%, range: 0.01–100%)*	<0.001		
⩽10	306 (42.2%)	96 (27.4%)	
10–50	361 (49.8%)	206 (58.9%)	
>50	58 (8.0%)	48 (13.7%)	
*Adjuvant radiation*			0.060
No	444 (61.2%)	194 (55.4%)	
Yes	281 (38.8%)	156 (44.6%)	
*Number positive nodes*			<0.001^b^
Mean	2.0	4.2	
Median	1	1	
Range	1–16	1–82	
*Total number of nodes examined*			<0.001^b^
Mean	15.8	20.6	
Median	14	16	
Range	1–88	1–90	
*Ratio of positive nodes*			0.001^b^
Mean	21.5%	26.8%	
Median	12.5%	18.2%	

a Marital Status: total *N*=1054 due to unknowns; not married includes single, divorced, separated, and widowed.

b *P*-values are based on independent sample *t*-test.

**Table 4:** Multivariate analysis for prognostication for disease-specific survival for stage IIIC endometrioid uterine cancer (*n*=1075)

**Factor**	**Hazard ratio**	**95% confidence interval**	***P*-value**
Age of diagnosis^a^	1.01	1.00–1.02	0.170
Grade^b^	2.19	1.62–2.96	<0.001
Location of LN^c^	1.27	0.87–1.84	0.218
Number of positive nodes^d^	1.04	1.00–1.08	0.065
Number of LN examined^e^	0.99	0.97–1.01	0.507
Ratio of positive nodes^f^	3.10	1.41–6.81	0.005
Adjuvant radiation^g^	0.47	0.33–0.66	<0.001
Marital status^h^	0.63	0.44–0.90	0.010

Abbreviation: LN=lymph node.

a Age at diagnosis as a continuous variable.

b Grade as 1 *vs* 2 *vs* 3 (undetermined grade excluded).

c Location of positive nodes as pelvic *vs* paraaortic with or without pelvic.

d Number of positive lymph nodes as a continuous variable.

e Number of lymph nodes examined as a continuous variable.

f Ratio of positive lymph nodes as a continuous variable.

g No adjuvant radiation *vs* administration of adjuvant radiation.

h Not married (including single, divorced, widowed, separated) *vs* married.

**Table 5:** Cox regression models for disease-specific survival for subgrouping by location, number, and ratio of positive lymph nodes in stage IIIC endometrioid uterine cancer patients

**Stage**	**Hazard ratio**	**95% CI**	**Number of positive nodes**	**Hazard ratio**	**95% CI**	**Ratio of positive nodes**	**Hazard ratio**	**95% CI**
IIIC 1	1	—	1	1	—	⩽23.2%	1	—
IIIC 2	1.72	1.23–2.41	>1	1.15	0.82–1.60	>23.2%	2.20	1.58–3.06
Continuous variable	NA	NA	Continuous variable	1.04	1.02–1.06	Continuous variable	4.47	2.66–7.54

Abbreviations: CI=confidence interval; NA=not applicable.
