# Supplementary material for: Prognostic discrimination of subgrouping node-positive endometrioid uterine cancer: location vs nodal extent
Source: Br J Cancer. 2011 Sep 13;105(8):1137–43. doi: 10.1038/bjc.2011.336 (PMC3208487; doi:10.1038/bjc.2011.336)
Supplement: Supplementary Information [file bjc2011336x1.doc]

Supplemental Table 1: Demographic and Clinical Characteristics for Subset of Stage IIIC Endometrioid Uterine Cancer Patients with Only One Positive Lymph Node (n=487)

| Parameters (average, range) | N(%) |
| --- | --- |
| Age at Diagnosis (63, 32-94) |  |
| <63 years | 239 (49.1%) |
| ≥63 years | 248 (50.9%) |
| Marital Status ^a^ |  |
| Married | 251 (52.4%) |
| Not Married | 228 (47.6%) |
| Race |  |
| White | 422 (86.7%) |
| Black | 29 (6.0%) |
| Asian | 33 (6.8%) |
| Unknown or Other | 3 (0.6%) |
| Grade |  |
| 1 | 83 (17.0%) |
| 2 | 195 (40.0%) |
| 3 | 168 (34.5%) |
| Unknown | 41 (8.4%) |
| Location of Positive Regional Nodes |  |
| Pelvic | 398 (81.7%) |
| Para-aortic without pelvic | 89 (18.3%) |
| Total Number of Nodes Examined (15.0, 1-90) |  |
| ≤10 | 205 (42.1%) |
| 11-20 | 150 (30.8%) |
| >20 | 132 (27.1%) |
| Ratio of Positive Nodes (17%, 0.01%-100%) |  |
| ≤average | 384 (78.9%) |
| >average | 103 (21.1%) |
| Ratio of Positive Nodes (23%, 0.01%-100%) |  |
| ≤10% | 301 (61.8%) |
| 10-50% | 158 (32.4%) |
| >50% | 28 (5.7%) |
| Adjuvant Radiation |  |
| No | 185 (38.0%) |
| Yes | 302 (62.0%) |

***** Median number of nodes examined = 12

1. Marital Status: total N=479 due to unknowns; not married includes single, divorced, separated, and widowed

Supplemental Table 2: Multivariate Analysis for Prognosticators in the Subset of Patients with Only One Positive Lymph Node (n=487)

| Factor | Hazard Ratio | 95% Confidence Interval | *P*-value |
| --- | --- | --- | --- |
| Age of diagnosis ^a^ | 1.02 | 0.995-1.04 | 0.122 |
| Marital Status ^b^ | 0.58 | 0.33-1.04 | 0.065 |
| Grade ^c^ | 2.54 | 1.59-4.07 | <0.001 |
| Number of LN examined^d^ | 0.99 | 0.95-1.03 | 0.612 |
| Ratio of Positive Nodes ^e^ | 2.91 | 1.03-8.18 | 0.043 |
| Adjuvant Radiation ^f^ | 0.45 | 0.26-0.78 | 0.004 |

***** Median number of nodes examined = 12

a Age at diagnosis as a continuous variable

b Not Married (including single, divorced, widowed, separated) vs. Married

c Grade as 1 vs. 2. vs. 3 (undetermined grade excluded)

dNumber of lymph nodes examined as a continuous variable

eRatio of positive nodes as a continuous variable

f No adjuvant radiation vs. administration of adjuvant radiation

Supplemental Table 3: Disease-Specific Survival Rates Comparing Substaging of Stage IIIC Endometrioid Uterine Cancer Patients by Location of Nodes, Number of Positive Nodes and Ratio of Positive to Total Number of Nodes

|  | Location | | |  | No. of Positive Nodes | | |  | Ratio of Positive Nodes | | |
| --- | --- | --- | --- | --- | --- | --- | --- | --- | --- | --- | --- |
|  | 1 year | 2 year | 3 year |  | 1 year | 2 year | 3 year |  | 1 year | 2 year | 3 year |
| All patients | 94.1 | 85.6 | 76.3 | All patients | 94.1 | 85.6 | 76.3 | All Patients | 94.1 | 85.6 | 76.3 |
| IIIC 1 | 94.9 | 88.0 | 80.5 | 1 | 94.0 | 85.4 | 79.5 | ≤23% | 97.1 | 89.5 | 80.8 |
| IIIC 2 | 92.5 | 80.3 | 67.0 | >1 | 94.3 | 85.7 | 73.4 | >23% | 88.0 | 78.3 | 67.6 |
| Separation* | 2.4 | 7.7 | 13.5 | Separation* | 0.3 | 0.3 | 6.1 | Separation* | 9.1 | 11.2 | 13.2 |
| Variability^#^ | 1.6 | 16.925 | 52.065 | Variability^#^ | 0.025 | 0.025 | 9.325 | Variability^#^ | 23.105 | 34.25 | 47.97 |

* Separation is the difference between survival rates of the two

subdivisions

# Variability is the average squared deviations of the two survival rates

from the overall population
